# The myofibroblast, multiple origins for major roles in normal and pathological tissue repair

**DOI:** 10.1186/1755-1536-5-S1-S5

**Published:** 2012-06-06

**Authors:** Ludovic Micallef, Nicolas Vedrenne, Fabrice Billet, Bernard Coulomb, Ian A Darby, Alexis Desmoulière

**Affiliations:** 1 Facultés de Médecine et de Pharmacie, Université de Limoges, EA 6309 "Maintenance Myélinique et Neuropathies Périphériques", FR 3503, Limoges F-87025, France; 2 Inserm U970, Réparation Artérielle, PARCC-HEGP, Université Paris Descartes, Paris, F-75015, France; 3 Cancer and Tissue Repair Laboratory, School of Medical Sciences, RMIT University, Bundoora, Victoria 3083, Australia

## Abstract

Myofibroblasts differentiate, invade and repair injured tissues by secreting and organizing the extracellular matrix and by developing contractile forces. When tissues are damaged, tissue homeostasis must be re-established, and repair mechanisms have to rapidly provide harmonious mechanical tissue organization, a process essentially supported by (myo)fibroblasts. Under physiological conditions, the secretory and contractile activities of myofibroblasts are terminated when the repair is complete (scar formation) but the functionality of the tissue is only rarely perfectly restored. At the end of the normal repair process, myofibroblasts disappear by apoptosis but in pathological situations, myofibroblasts likely remain leading to excessive scarring. Myofibroblasts originate from different precursor cells, the major contribution being from local recruitment of connective tissue fibroblasts. However, local mesenchymal stem cells, bone marrow-derived mesenchymal stem cells and cells derived from an epithelial-mesenchymal transition process, may represent alternative sources of myofibroblasts when local fibroblasts are not able to satisfy the requirement for these cells during repair. These diverse cell types probably contribute to the appearance of myofibroblast subpopulations which show specific biological properties and which are important to understand in order to develop new therapeutic strategies for treatment of fibrotic and scarring diseases.

## Introduction

Tissue repair is an essential phenomenon allowing tissues and organs to recover functional properties that have been lost after an injury, either linked to a wound or to a disease. Contrary to what is seen in fetal or embryonic wounds that repair without a scar or fibrosis, normal repair in the adult always leads to scar formation, the consequence of which may be defects in functionality (e.g. skin hypertrophic scar or fibrosis). In these processes, fibroblasts/myofibroblats play a crucial role. Moreover, myofibroblasts are instrumental in the stroma reaction to epithelial tumors and are now thought to promote cancer progression by creating a stimulating microenvironment for the transformed cells [1,2].

## The myofibroblast in normal and pathological situations

### Normal wound healing

**Figure 1:**
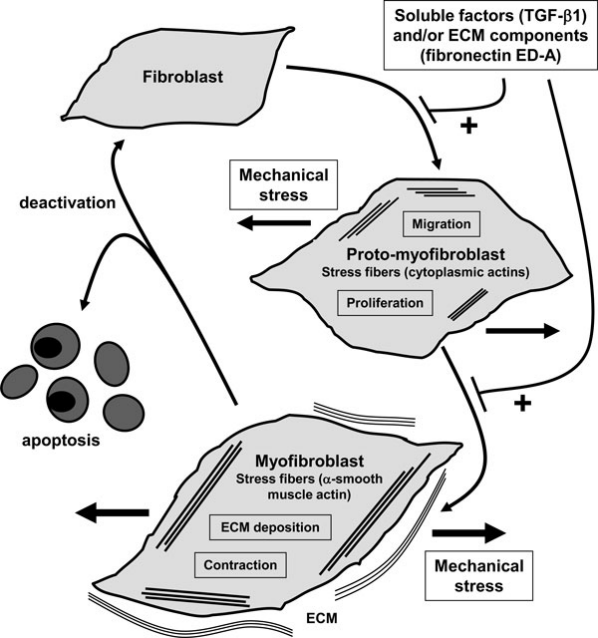
**Schematic illustration showing the evolution of the (myo)fibroblast phenotype**. The myofibroblastic modulation of fibroblastic cells begins with the appearance of the proto-myofibroblast, whose stress fibers contain only β- and γ-cytoplasmic actins and evolves, but not necessarily always, into the appearance of the differentiated myofibroblast, the most common variant of this cell, with stress fibers containing α-smooth muscle actin. The myofibroblast may disappear by apoptosis; the deactivation leading to a quiescent phenotype has not been clearly demonstrated at least *in vivo*. TGF-β1: transforming growth factor-β1; ECM: extracellular matrix. Modified from [38].

Immediately after wounding, the healing process allowing restoration of injured tissue occurs. Wound healing proceeds in three interrelated dynamic phases with overlapping time courses. According to morphological changes in the course of the healing process, these phases are described as an inflammatory phase, a proliferative phase for the development of granulation tissue, and a regeneration phase for maturation, scar formation and re-epithelialisation [3]. The inflammatory phase begins with damage to the capillaries, which triggers the formation of a blood clot consisting of fibrin and fibronectin. This provisional matrix will fill in the lesion and will allow the various recruited cells to migrate into wound. Platelets present in the blood clot release multiple chemokines which participate in the recruitment not only of inflammatory cells (neutrophils and macrophages), but also fibroblasts and endothelial cells. The second stage of wound healing is the proliferative phase. Active angiogenesis which is critical for the wound healing process, allows new capillaries to deliver nutrients including oxygen to the wound, and contributes to the proliferation of fibroblasts. In the granulation tissue, fibroblasts become activated and acquire a smooth muscle cell-like phenotype; they are consequently called myofibroblasts. These myofibroblastic cells synthesize and deposit the extracellular matrix components which will replace the provisional matrix. These cells also exhibit contractile properties, due to the expression of α-smooth muscle actin in microfilament bundles or stress fibers, playing a major role in the contraction and in maturation of the granulation tissue [4] (Figure 1). Presently, it is accepted that the myofibroblastic modulation of fibroblastic cells begins with the appearance of the protomyofibroblast, whose stress fibers contain only β- and γ-cytoplasmic actins. Protomyofibroblasts evolve in most cases into differentiated myofibroblasts, the most common variant of this cell, with stress fibers containing α-smooth muscle actin (for review, see [5]) (Figure 1). Myofibroblasts can, depending on the experimental or clinical situation, express other smooth muscle cell specific contractile proteins, such as smooth muscle-myosin heavy chains or desmin; however, the presence of α-smooth muscle actin represents the most reliable marker of the myofibroblastic phenotype [6]. The third phase of healing, scar formation, involves a progressive remodelling of the granulation tissue. During this remodelling process, proteolytic enzymes, essentially matrix metalloproteinases (MMPs) and their inhibitors (TIMPs for tissue inhibitor of metalloproteinases) play a major role [7]. In the resolution phase of healing, the cell number of vascular cells and myofibroblasts is dramatically reduced by the process of apoptosis [8] (Figure 1). To date, it has not been clearly shown that myofibroblasts can reacquire a quiescent phenotype.

### Pathological repair

Pathological wound healing can be encountered in a variety of disease states [9]. These abnormal repair processes are the result of an impaired remodelling of the granulation tissue leading for example to abnormal cutaneous repair as seen in hypertrophic scarring or to fibrosis in internal organs such as the liver, lung and kidney. In hypertrophic scars, numerous myofibroblasts express α-smooth muscle actin, explaining the frequent appearance of contracture [9]. In internal organs, after an acute and moderate lesion, the injured tissue may be almost completely restored to normal. The repair process involved is globally similar to the process observed in cutaneous wounding. However, when the noxious stimulus responsible for the lesion persists, excessive extracellular matrix deposition and the continued presence of myofibroblasts is observed. This excess of extracellular matrix deposition leads to the development of organ fibrosis. For example, in the liver, several chronic diseases (chronic viral hepatitis, alcoholic disease and cholestasis) are responsible for the development of a significant fibrosis whose ultimate stage, cirrhosis, has a substantial impact on morbidity and mortality. As in pathological cutaneous wound healing, the installation and persistence of fibrosis is the consequence of an imbalance between extracellular matrix synthesis and degradation by myofibroblasts. In this situation the balance of MMPs/TIMPs plays an essential role. For example, throughout hepatic fibrogenesis, an increase of TIMP-1 and TIMP-2 expression without any modification of MMP-1 is observed and it is thought that this leads to excessive matrix deposition.

### Cytokines involved in myofibroblast differentiation

Various cytokines and growth factors have a role in wound healing and scarring [10]. Among these soluble factors some directly act on granulation tissue formation and fibrogenic cell activation, especially transforming growth factor (TGF)-β1, a potent inducer of myofibroblastic differentiation [11]. Beyond a specific effect on the induction of α-smooth muscle expression, TGF-β1 also promotes the deposition of large amounts of extracellular matrix; in fact, TGF-β1 not only induces synthesis of extracellular matrix, particularly fibrillar collagens and fibronectin but it also reduces MMP activity by promoting TIMP expression. It is important to note that TGF-β1 action on myofibroblastic differentiation is only possible in the presence of the ED-A splice variant fibronectin which underlines the fact that extracellular matrix components play an important role in soluble factor activity [12]. More recently, it has been shown that granulation tissue formation is modified by chemical denervation [13]. This peripheral nervous system involvement in tissue repair has likewise been shown in the liver, where in this organ, in an experimental model of fibrosis using carbon tetrachloride treatment, chemical denervation significantly reduces matrix deposition and myofibroblastic differentiation [14].

### Role of mechanical stress

Myofibroblastic cells, because of their contractile properties and their privileged relationship with the extracellular matrix, can modify their activity depending on the mechanical environment. Although this is an essential point, it still remains poorly investigated. It has been shown, in gingival fibroblasts, that α-smooth muscle actin expression induced by TGF-β1 is regulated by the compliance of collagen gels on or in which they are cultured [15]. Moreover, myofibroblastic differentiation features, such as stress fibers, ED-A fibronectin or α-smooth muscle expression, appear earlier in granulation tissue subjected to an increase in mechanical tension by splinting of a full-thickness wound with a plastic frame as compared to normally healing wounds [16]. It has also been shown that fibroblasts cultured on substrates of variable stiffness present different phenotypes. Cultured fibroblasts do not express stress fibers on soft surfaces; whilst when the stiffness of the substrate increases, a sudden change in cell morphology occurs and stress fibers appear [17,18]. More recently, it has been shown that shear forces exerted by fluid flow are also able to induce TGF-β1 production and differentiation of fibroblasts cultured in collagen gels in the absence of other external stimuli such as cytokine treatment [19].

## Origin of the myofibroblast

**Figure 2:**
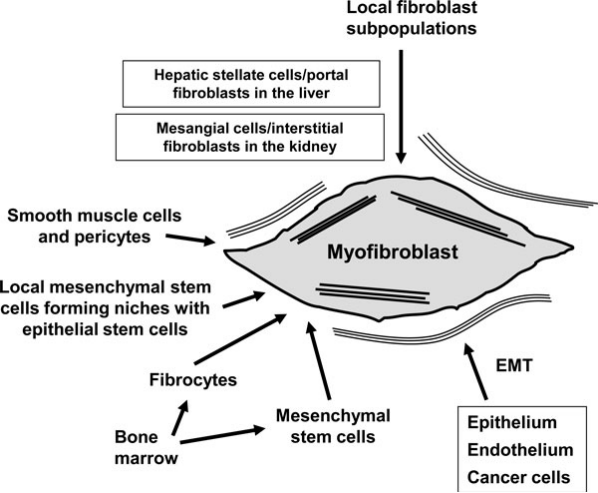
**Myofibroblast origins**. The main myofibroblast progenitor after injury of different tissues appears to be locally residing fibroblasts. Indeed, various cell types can acquire a myofibroblastic phenotype; these diverse origins lead to distinct myofibroblast sub-populations. EMT: epithelial- and endothelial-to-mesenchymal transition.

Myofibroblasts can originate from various cell types as illustrated in Figure 2. The major contribution of the cells originates from local recruitment of connective tissue fibroblasts. For example in the skin, dermal fibroblasts located at the edges of the wound can acquire a myofibroblastic phenotype and participate in tissue repair. In the liver, the role of perisinusoidal hepatic stellate cells has been widely studied and their key role during fibrogenesis has been clearly demonstrated. However, in the connective tissue, important heterogeneity in fibroblastic cell subpopulations has been observed. These subpopulations reside in different locations within the organ and have specific activation and deactivation properties. At least three subpopulations have been identified in the dermis: superficial dermal fibroblasts, reticular fibroblasts, which reside in the deep dermis, and fibroblasts associated with hair follicles. These cell subpopulations can be isolated and exhibit distinctive differences when cultured separately [20]. In the liver, in the main two subpopulations of fibrogenic cells have been described: i) the hepatic stellate cells located in the space of Disse between the hepatocytes and the sinusoidal endothelial cells and ii) the portal fibroblasts located in the connective tissue surrounding portal tracts [21]. We recently applied the precision cut-liver slice (PCLS) model which preserves the normal lobular architecture and allows the maintenance of cell-cell interactions within their original extracellular matrix to study hepatic stellate cell and portal fibroblast behaviour in rat PCLS derived from fibrotic tissue [22,23]. In pathological situations, myofibroblastic cells expressing α-smooth muscle actin are derived either from hepatic stellate cells (e.g. in alcoholic cirrhosis where myofibroblasts are present in the parenchyma and in septa) or from portal fibroblasts (e.g. in cholestatic fibrosis where myofibroblasts are present in enlarged portal areas). Thus, in the liver, fibrogenic cells mainly include hepatic stellate cells and portal fibroblasts, even if, in some cases, second layer cells located around centrilobular veins can be involved in parenchymal fibrosis [24]. Moreover, today, the involvement in tissue repair of local mesenchymal stem cells is discussed more and more. These progenitor cells have been described in the dermal sheath that surrounds the outside of the hair follicle facing the epithelial stem cells. They are involved in the regeneration of the dermal papilla and they can also became wound healing (myo)fibroblasts after a lesion [25]. Mesenchymal stem cells have not yet been described in the liver, but at the periphery of portal tracts, in Hering's canals, epithelial stem cells have been described. Hering's canal represents the junction between hepatocytes and bile duct epithelial cells, and in this area, numerous proliferative cells are observed after a hepatic lesion. This area may constitute a niche containing not only epithelial stem cells but also mesenchymal stem cells and these two cell types may cooperate depending on the tissue need. This concept of a cell association, able to reconstitute the different organ cell populations and constituting a niche of stem cells is currently discussed in diverse organs notably in the liver [26]. Recent data have also implicated circulating cells, called fibrocytes, in the tissue repair process. Fibrocytes enter into injured skin together with inflammatory cells and acquire a myofibroblastic phenotype [27]. In post-burn scars, fibrocytes are recruited to the site of the lesion where they stimulate local inflammatory response and produce extracellular matrix proteins thus contributing to hypertrophic scar formation [28]. Fibrocytes are also implicated in the lung in subepithelial fibrosis observed in asthma [29], and in renal fibrosis [30]. Another type of circulating cell originating from bone marrow has been suggested to participate in tissue repair. These mesenchymal stem cells are bone marrow-derived non-hematopoietic precursor cells [31] that contribute to the maintenance and regeneration of connective tissues through engraftment. Indeed, they have the capacity to engraft into several organs and to differentiate into wound healing myofibroblasts. The engraftment in injured organs is regulated by the severity of the damage [32]. Interestingly, very poor engraftment of intravenously administered mesenchymal stem cells in healthy organs was observed. In hepatic fibrosis, a significant proportion of myofibroblasts may originate from the bone marrow [33]. Finally, epithelial- and endothelial-to-mesenchymal transition (EMT), a process by which differentiated or malignant epithelial and endothelial cells undergo a phenotypic conversion that gives rise to the matrix-producing fibroblasts and myofibroblasts, is increasingly recognized as an integral part of tissue fibrogenesis after injury, particularly in the kidney [34]. However, the degree to which this process contributes to fibrosis remains a matter of intense debate and is likely to be context-dependent. All together, mesenchymal stem cells, fibrocytes, bone marrow-derived cells and cells derived from an EMT process, may represent alternative sources of myofibroblasts when local fibroblasts are not able to satisfy the tissue's requirement for these cells.

## Conclusions and perspectives

The fibroblast/myofibroblast transition is accepted as the key event in the formation of granulation tissue during wound healing or fibrotic changes, but also during the evolution of the stroma reaction in cancer. Though different experimental models have been developed, until now, the exact origin of the (myo)fibroblastic cells involved in the formation of the stroma reaction observed in carcinomas is unknown. Obviously, local fibroblasts present in the connective tissue of the organ are involved. However, it has also been shown that bone marrow-derived myofibroblasts contribute to the stroma reaction [35,36]. Interestingly, the question arises as to why the stroma reaction is scanty in hepatocellular carcinoma but abundant in cholangiocarcinoma. It is conceivable that the cells contributing to the myofibroblastic population and which participate in the stroma reaction are different in these two tumors. We suggest that hepatic stellate cell-derived myofibroblasts and portal fibroblast-derived myofibroblasts are involved in the stroma reactions encountered in hepatocellular carcinoma and cholangiocarcinoma, respectively, and we have recently performed a proteomic study in order to make progress in this field [37]. All of this information is required for the development of treatments that could specifically and efficiently target the cells responsible for the development of fibrotic diseases and of the stroma reaction in cancers. Indeed, stroma-myofibroblast interactions represent an interesting tumor differentiation-independent target for therapy of cancers, particularly for hepatocellular carcinoma and cholangiocarcinoma which are highly aggressive cancers.

## List of abbreviations used

EMT: epithelial- and endothelial-to-mesenchymal transition; MMP: matrix metalloproteinase; PCLS: precision cut-liver slice; TGF: transforming growth factor; TIMP: tissue inhibitor of metalloproteinases.

## Competing interests

The authors declare that they have no competing interests.

## Authors' contributions

LM, NV, and FB wrote the first draft of the manuscript. BC, IAD and AD provided constructive criticism, and contributed substantially to the final draft of the manuscript. AD was responsible for its design and coordination. All authors read and approved the final manuscript.

## Authors' information

BC, IAD, and AD have worked on mechanisms involved in tissue repair and fibroblast subpopulations for over 20 years. LM has developed with BC and AD, research on mesenchymal stem/stromal cells and their roles in tissue repair. NV and FB performed studies on matrix metalloproteinases and their inhibitors.
